# Structure Based Multitargeted Molecular Docking Analysis of Selected Furanocoumarins against Breast Cancer

**DOI:** 10.1038/s41598-019-52162-0

**Published:** 2019-10-31

**Authors:** Reetuparna Acharya, Shinu Chacko, Pritha Bose, Antonio Lapenna, Shakti Prasad Pattanayak

**Affiliations:** 1 Division of Advanced Pharmacology, Department of Pharm. Sciences & Technology, Birla Institute of Technology, Mesra, Ranchi, 835215 India; 2 Division of Pharmaceutical Chemistry, Department of Pharm. Sciences & Technology, Birla Institute of Technology, Mesra, Ranchi, 835215 Jharkhand India; 3 Department of Oncology and Metabolism, University of Sheffield Medical School, Beech Hill Road, Sheffield, S102RX United Kingdom; 4 Research Manager, Clinical Pharmacology and Pharmacokinetics, Sun Pharmaceutical Industries Limited, Gurgaon, 122015 India

**Keywords:** Cancer, Breast cancer, Breast cancer

## Abstract

Breast cancer is one of the biggest global dilemmas and its current therapy is to target the hormone receptors by the use of partial agonists/antagonists. Potent drugs for breast cancer treatment are Tamoxifen, Trastuzumab, Paclitaxel, etc. which show adverse effects and resistance in patients. The aim of the study has been on certain phytochemicals which has potent actions on ERα, PR, EGFR and mTOR inhibition. The current study is performed by the use of molecular docking as protein-ligand interactions play a vital role in drug design. The 3D structures of ERα, PR, EGFR and mTOR were obtained from the protein data bank and docked with 23 3D PubChem structures of furanocoumarin compounds using FlexX. Drug-likeness property was checked by applying the Lipinski’s rule of five on the furanocoumarins to evaluate anti-breast cancer activity. Antagonist and inhibition assay of ERα, EGFR and mTOR respectively has been performed using appropriate *in-vitro* techniques. The results confirm that Xanthotoxol has the best docking score for breast cancer followed by Bergapten, Angelicin, Psoralen and Isoimperatorin. Further, the *in-vitro* results also validate the molecular docking analysis. This study suggests that the selected furanocoumarins can be further investigated and evaluated for breast cancer treatment and management strategies.

## Introduction

Breast cancer is one of the most common types of malignancies in women worldwide. Breast carcinogenesis is unrecognized because of a variety of risk factors in context to bio-molecular dynamics. The risk of breast cancer has increased since the past 50 years and accounts for 23% of all cancer deaths in Asia according to the statistical reports of WHO (2012)^1^. Cancer is caused due to genetic damage in cells which show faulty division and mutation. Breast cancer is a type of hormonal cancer. Breasts contain glandular tissues which are highly sensitive to hormonal changes in the body^2^.

During embryogenesis, mammary gland development begins which proceeds with further growth and differentiation during puberty and pregnancy. The female sex hormones, estradiol and progesterone cause embryonic enlargement and growth of breast ducts, ductules and buds. The concentration of estrogen is higher during puberty which augments estrogen and progesterone receptor synthesis in mammary glands. These sex hormones create potential risk factors for the development of breast cancer^3^. On the other hand, pregnancy and lactation help in reducing the cancer development risk. Studies show that higher breast mass is associated with higher risks of developing breast malignancies^4^. Higher breast mass is associated with increased number of fat cells which make estradiol from cholesterol that increases the risk of breast cancer. Studies report that the formation of estradiol from cholesterol is a key factor for the development of breast cancer in post-menopausal women due to the presence of excess of fat cells in the breasts. The other risk factors of breast cancer include childbirth after 30 years age, obesity, presence of benign tumors in the breast, vitamin D deficiency, lack of exposure to sunlight, etc^2^.

Breast cancer chemotherapy is marked by targeting the function of receptors such as ERα (estrogen receptor alpha), PR (progesterone receptor), EGFR (epidermal growth factor receptor) etc. Estrogen receptors (ER) play a vital role in the initiation and progression of breast cancer. Studies say that estrogen, specifically 17 β-estradiol has been reported to upregulate the expression and function of c-Myc and cyclinD1 which leads to the promotion of cell cycle from G1 phase to S phase in the epithelial cells of mammary glands. Anti-estrogen therapy is hereby a promising approach for the treatment of ER positive breast cancer and also the first targeted therapy for human breast cancer^5^. The over-expression of PR is usually observed in breast cancer and this is directly related to the over-expression of ER as PR is the end product from estrogenic stimulation in target tissues which indicated a functioning ER pathway. The over-expression of PR along with ER provides better prognosis for PR positive breast cancer and there are better chances of response to hormonal therapy^6^. Treatment with ER and PR antagonists can bring upon better treatment options and prognosis. EGFR has been reported to play an important role in triple negative breast cancer (TNBC)^7^. Several TNBC cell lines (MDA-MB-231 and MDA-MB-468) have been found to have an increased level of EGFR^8^. Since TNBC is phenotypically characterized as ER negative, PR negative and HER-2 negative (hence the name triple negative breast cancer), the treatment options are very narrow^9^. Hence, the use of EGFR partial agonists/antagonists can show promising treatment strategies. The approved marketed therapeutic drugs used for breast cancer are Tamoxifen, Trastuzumab, Paclitaxel, Capecitabine, Cyclophosphamide, Gemcitabine, Docetaxel etc. which have diversified side effects^10^. The other medicinal alternatives for the treatment of breast cancer are certain phytochemicals and their derivatives which have been proven to show potent anti-cancer action^10^.

Phytochemicals are regularly investigated for modern medicine nowadays. These compounds serve as a major factor for the synthesis of various therapeutic agents^11^. Phytochemicals have been reported to show various encouraging activities against human cancer models^12–15^. The phytochemicals are classified into isoflavones, coumarins, terpenoids, monoterpenes, etc. Coumarins tend to show extensive therapeutic activities including photochemotherapy, anti-neoplastic activity, anti-HIV, anti-coagulants, anti-bacterial, anti-inflammatory, CNS stimulants and dyes. The derivatives of coumarins are pyranocoumarins, furanocoumarins, coumarin sulfamates (coumates), etc^15^. Furanocoumarins are secondary metabolites obtained from higher plants usually belonging to the families, rutaceae and umbelliferae^16^. The furanocoumarins used for docking analysis in the current study are Xanthotoxol, Psoralen, Isoimperatonin, Bergapten, Angelicin, Visnagin, Isobergapten, Sphondin, Lanatin, Khellin, Xanthotoxin, Apterin, 6,7-dihydroxy bergamottin, Marmesin, Methoxsalen, Imperatonin, Isopimpinellin, Trioxsalen, Bergamottin and Phellopterin.

Molecular docking is a methodology applied to study molecular behavior on target proteins binding. It is a tool which is used extensively in drug discovery. The top software used for best scores in docking are AutoDock, Vina, MOE-Dock, FLexX and GOLD respectively. For predicting the correct binding poses, GOLD and LeDock are used^17^.

Taking into consideration, the role of certain target receptors i.e. ERα (Estrogen receptor), PR (Progesterone receptor), EGFR (Epidermal growth factor receptor) and mTOR (mammalian target of rapamycin) in the initiation and progression of breast cancer, we proposed 23 furanocoumarin compounds having anti-cancer potential. In the present study, we proposed to work on docking studies using BioSolveIT FlexX (2.3.1) to evaluate the anti-cancer activity of the concerned phytochemicals. FlexX is a fully automated docking tool that docks compounds into the enzymes’ active sites. FlexX has an excellent ligand flexibility which can be achieved by changing the conformations of the ligand in active site making the protein rigid^18^. FlexX uses incremental construction to build up the ligand within a binding site. It gives an all atom representation for the protein^19^. FlexX software is a very flexible and fast algorithm for small ligands docking in binding sites of receptors and enzymes^20^. FlexX also incorporates interactions between protein and ligand, ligand core placement, and complete ligand rebuilding^21^. The docking study is based on the hypothesis that the furanocoumarins are capable of interfering with the activities of above-mentioned target receptors and cause inhibition of their activity and cancer progression.

## Results

### Structure of target proteins

The main therapeutic targets for breast cancer taken for the study were ERα, PR, EGFR, and mTOR. The three-dimensional structures of the following breast cancer target proteins were availed from protein data bank with the PDB ids: 3ERT, 4OAR, 2J6M, and 4DRH respectively.

### Molecular docking using FlexX and Drug likeness

In the study, various furanocoumarin compounds available in protein data bank were docked and their binding affinities were evaluated with some common protein targets for breast cancer. The value was calculated as per the binding affinity energies.

The ligand confirmation of a total of 23 furanocoumarin compounds was done according to the binding affinities with the receptor targets ERα, PR, EGFR, and mTOR. The co-crystallized structures of 4-hydroxy tamoxifen (TAM), Ulipristal acetate, AEE788, and Rapamycin (RAP) which are active against breast cancer^22–25^ with the respective PDB IDs 3ERT, 4OAR, 2J6M and 4DRH were downloaded with scores 34.43 kcal/mol, −21.10 kcal/mol, −19.21 kcal/mol and −46.09 kcal/mol and these co-crystallized structures presented RMSD values of 0.7766 Å, 1.1922 Å, 1.1133 Å and 1.6347 Å respectively which are depicted in Table 1, Fig. S1.

**Table 1:** Binding affinity energies (in kcal/mol) and RMSD values (in Å) of potent compounds active against breast cancer available in Protein Data Bank (PDB).

Reference compounds available in PDB	Structures	Target receptors	Binding affinity (in kcal/mol)	RMSD values (in Å)
TAM		ERα	−34.43	0.7766
Ulipristal acetate		PR	−21.10	1.1922
AEE788		EGFR	−19.21	1.1133
RAP		mTOR	−46.09	1.6347

**Figure 1:**
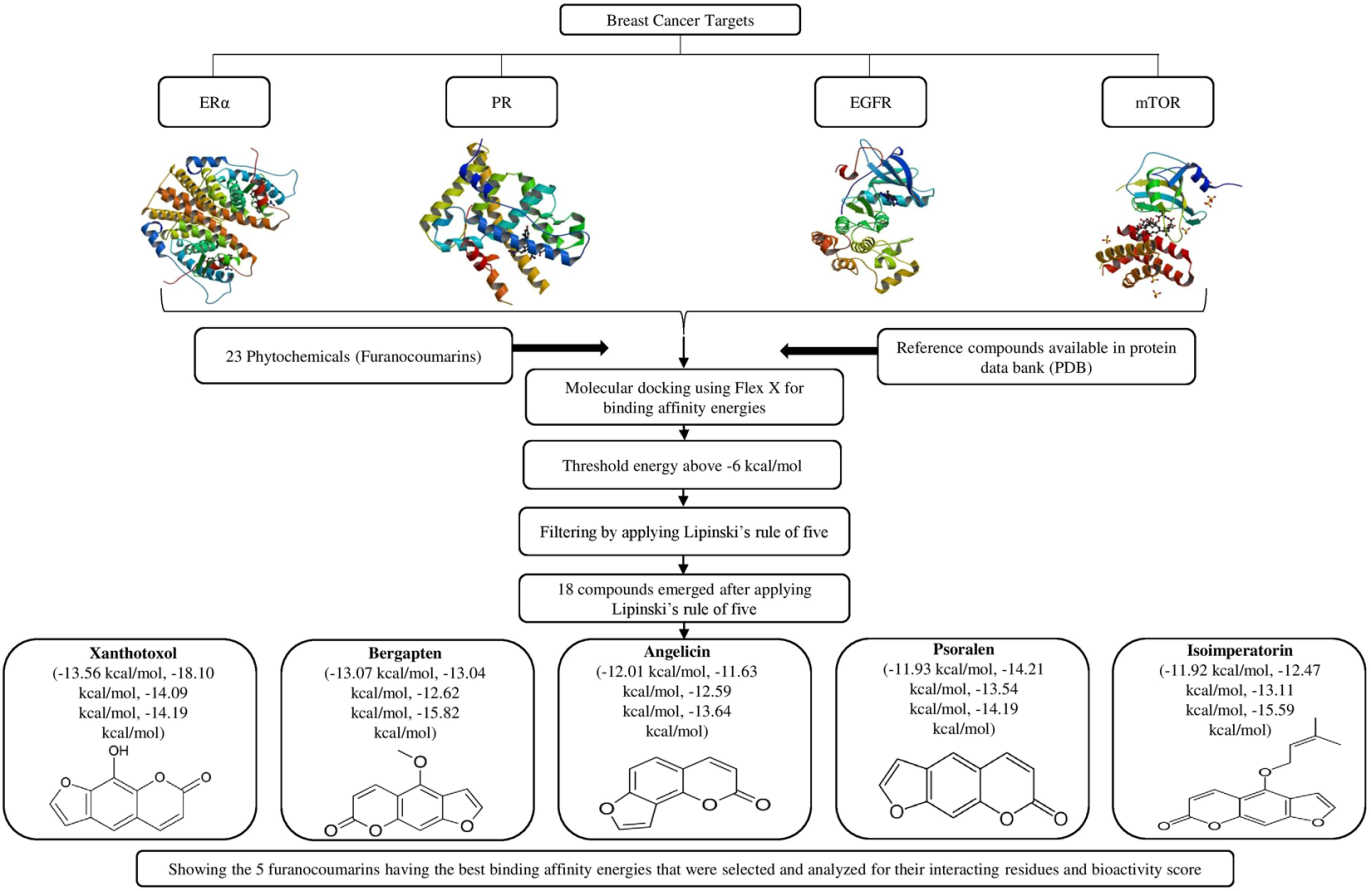
Representation of selection of compounds by different approaches used in the study.

Amongst the 23 furanocoumarins screened, 19 compounds exhibited docking energy values above −6 kcal/mol for breast cancer receptors, ERα, PR, EGFR and mTOR. Further, molecular properties of the furanocoumarin compounds were evaluated using Molinspiration to fit into the Lipinski rule of five, which is a key way to satisfy the rational drug design and calculate the bioactivity score for drugs meant for oral use (Molecular properties of furanocoumarin compounds are shown in Table 2). 20 out of 23 compounds were found to show no violations for the Lipinski rule of 5 i.e. not more than 5 hydrogen bond donors, not more than 10 hydrogen bond acceptors, partition coefficient not more than 5 (log P), rotatable bonds less than 10, total polar surface area not more than 140 and molecular weight less than 500 g/mol. A flow chart of different analyses applied to the furanocoumarin compounds for the best selection is shown in Fig. 1.

**Table 2:** (a) List of phytochemicals shortlisted by implementing Lipinski’s rule of five and their Molinspiration bioactivity details.

Phytochemicals	miLogP	TPSA	natoms	MW	nON	nOHNH	nviolation	nrotb	volume
XAN	2.00	63.58	15	202.16	4	1	0	0	162.16
BER	2.28	52.59	16	216.19	4	0	0	1	179.69
ANG	2.29	43.35	14	186.17	3	0	0	0	154.15
PSO	2.29	43.35	14	186.17	3	0	0	0	154.15
IMP	3.95	52.59	20	270.28	4	0	0	3	240.47
Phytochemicals	GPCR ligand	Ion channel modulator	Kinase inhibitor	Nuclear receptor ligand	Protease inhibitor	Enzyme inhibitor			
XAN	−0.70	−0.16	−0.82	−0.75	−0.94	−0.14			
BER	−0.65	−0.07	−0.98	−1.14	−0.98	−0.27			
ANG	−0.87	−0.48	−0.88	−0.93	−1.15	−0.28			
PSO	−0.89	−0.38	−1.10	−1.13	−1.19	−0.37			
IMP	−0.35	0.02	−0.73	−0.45	−0.65	−0.00			

(b) Molinspiration bioactivity score of selected furanocoumarin compounds.

The top five furanocoumarins that exhibited the highest binding affinity energies towards ERα, PR, EGFR and mTOR were XAN, BER, ANG, PSO and IMP. In these top five evaluations, XAN was found to show the best docking confirmation with binding affinity of −13.56 kcal/mol towards ERα, −18.10 kcal/mol towards PR, −14.09 kcal/mol for EGFR and −11.93 kcal/mol for mTOR closely followed by BER, ANG, PSO, and IMP with binding affinities of 13.07 kcal/mol, −12.01 kcal/mol, −11.93 kcal/mol, −11.92 kcal/mol towards ERα, −13.04 kcal/mol, −11.63 kcal/mol, −14.21 kcal/mol and −12.47 kcal/mol towards PR, −12.62 kcal/mol, −12.59 kcal/mol, −13.54 kcal/mol and −13.11 kcal/mol towards EGFR and −15.82 kcal/mol, −13.64 kcal/mol, −14.19 kcal/mol and −15.69 kcal/mol towards mTOR respectively as shown in Table 3.

**Table 3:** Binding affinity energies (in kcal/mol) of shortlisted furanocoumarin compounds against ERα, PR, EGFR and mTOR as cancer target sites.

Phytochemicals (Furanocoumarins)	Structure	Binding affinity (in kcal/mol)			
		ERα	PR	EGFR	mTOR
XAN		−13.556	−18.1096	−14.0949	−12.4596
BER		−13.0723	−13.0489	−12.6202	−15.8240
ANG		−12.0107	−11.6317	−12.5955	−13.6447
PSO		−11.9384	−14.2120	−13.5405	−14.1905
IMP		−11.9196	−12.4716	−13.1183	−16.6907

**Figure 2:**
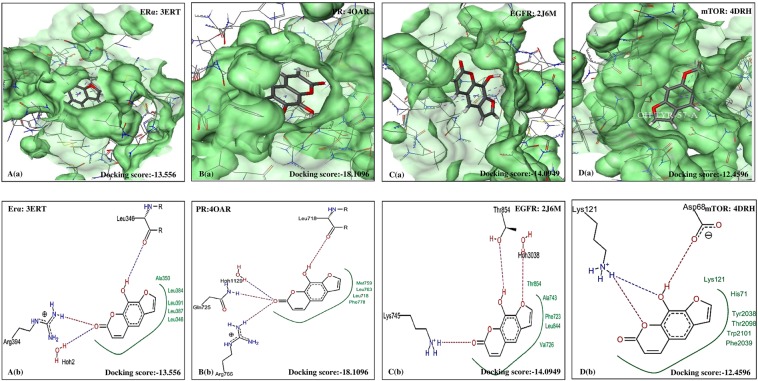
Molecular docking analysis of XAN. (**a**) Pose view of interaction of Xanthotoxol with receptors ERα, PR, EGFR and mTOR. (**b**) Overlay of XAN in active pockets of ERα, PR, EGFR and mTOR. XAN: Xanthotoxol, ERα: Estrogen receptor, PR: Progesterone receptor, EGFR: Epidermal growth factor receptor and mTOR: Mammalian target of Rapamycin.

**Figure 3:**
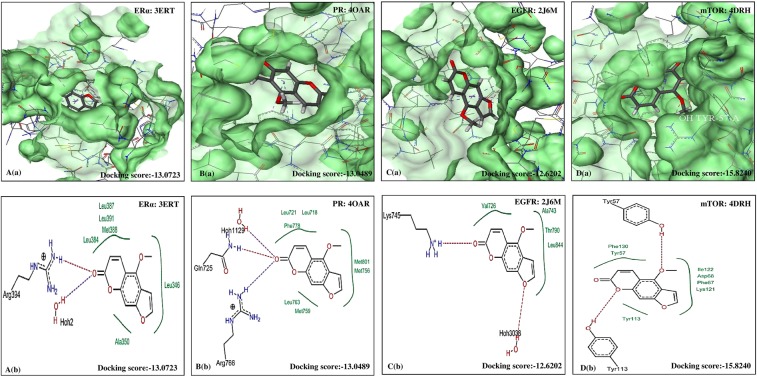
Molecular docking analysis of BER. (**a**) Pose view of interaction of BER with receptors ERα, PR, EGFR and mTOR. (**b**) Overlay of BER in active pockets of ERα, PR, EGFR and mTOR. BER: Bergapten, ERα: Estrogen receptor, PR: Progesterone receptor, EGFR: Epidermal growth factor receptor and mTOR: Mammalian target of Rapamycin.

**Figure 4:**
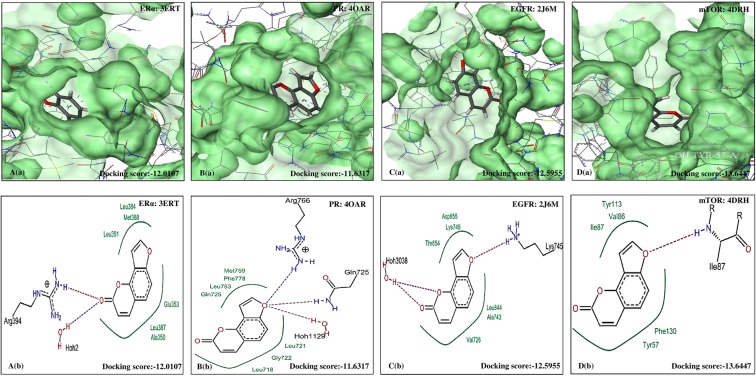
Molecular docking analysis of ANG. (**a**) Pose view of interaction of ANG with receptors ERα, PR, EGFR and mTOR. (**b**) Overlay of ANG in active pockets of ERα, PR, EGFR and mTOR. ANG: Angelicin, XAN: Xanthotoxol, ERα: Estrogen receptor, PR: Progesterone receptor, EGFR: Epidermal growth factor receptor and mTOR: Mammalian target of Rapamycin.

**Figure 5:**
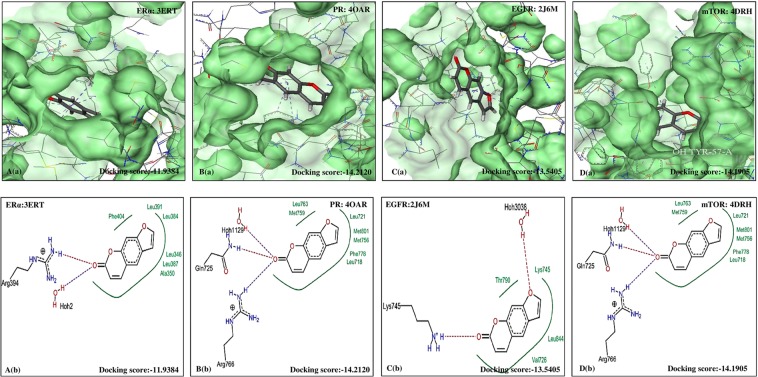
Molecular docking analysis of PSO. (**a**) Pose view of interaction of PSO with receptors ERα, PR, EGFR and mTOR. (**b**) Overlay of PSO in active pockets of ERα, PR, EGFR and mTOR. PSO: Psoralen, ERα: Estrogen receptor, PR: Progesterone receptor, EGFR: Epidermal growth factor receptor and mTOR: Mammalian target of Rapamycin.

**Figure 6:**
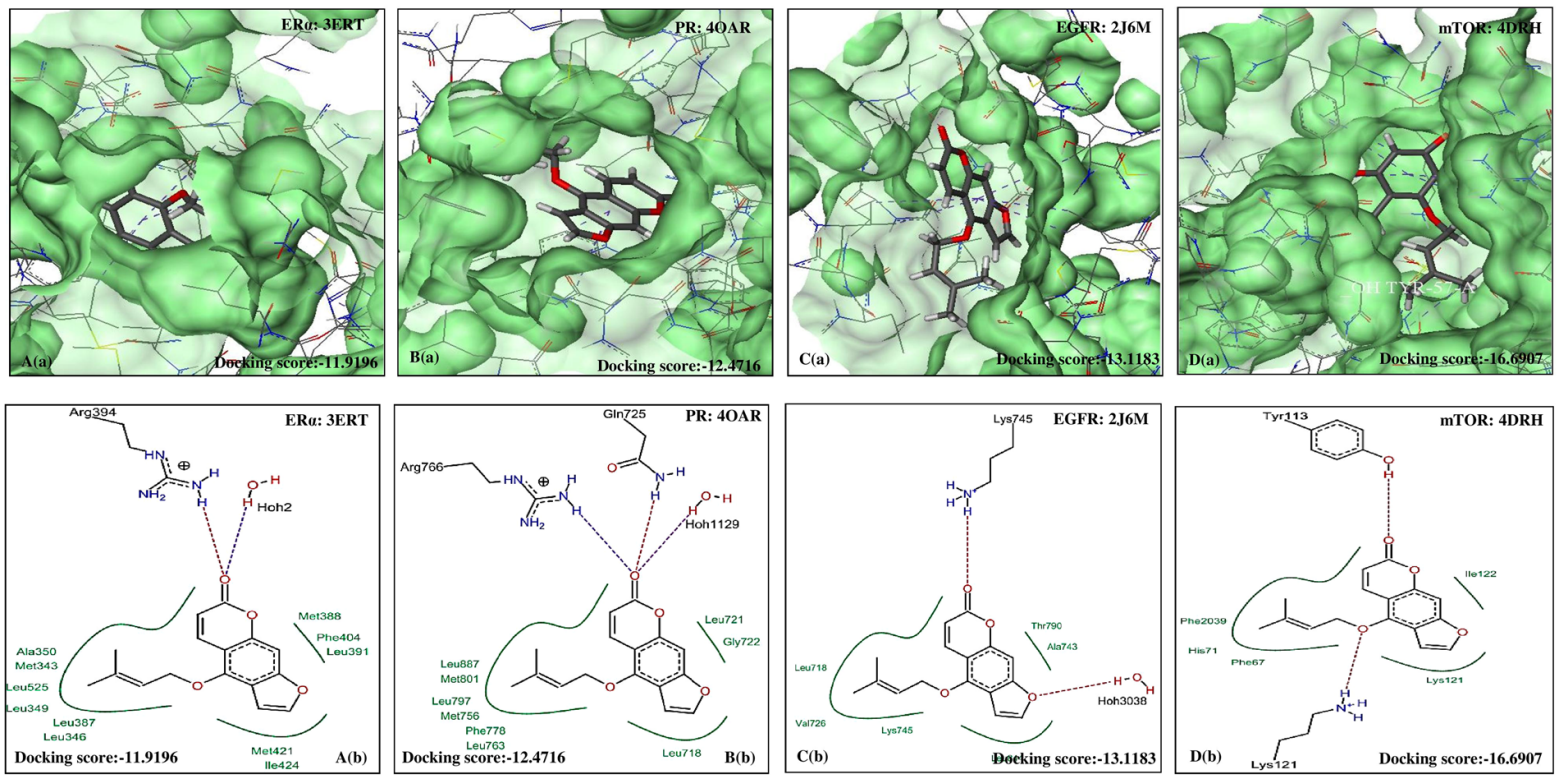
Molecular docking analysis of ISO. (**a**) Pose view of interaction of ISO with receptors ERα, PR, EGFR and mTOR. (**b**) Overlay of ISO in active pockets of ERα, PR, EGFR and mTOR. IMP: Isoimperatorin, ERα: Estrogen receptor, PR: Progesterone receptor, EGFR: Epidermal growth factor receptor and mTOR: Mammalian target of Rapamycin.

The five furanocoumarins viz. XAN, PSO, IMP, BER and ANG were seen to exhibit good bioactivity properties and drug-likeness as shown in Table 4. The best docking pose was also identified to reveal the most interacting residues in the active sites of ERα, PR, EGFR and mTOR. The interactions between the selected furanocoumarins and the four individual therapeutic targets showing the pose view and with their active pockets are shown in Figs 2–6.

**Table 4:** Amino acids interaction (hydrophobic binding and polar H binding) with the specific receptors of ERα, PR, EGFR and mTOR.

Selected bio-molecules	Protein and PDB IDs	Docking score (Ref/selected bio-molecules)	Nature of interactions	Amino acids on active sites with
XAN	EGFR (2J6M)	−19.2199/−14.0949	Hydrophobic interaction	Thr854, Ala743, Phe723, Leu844, Val726.
			Polar H interactions	Thr854, Lys745, Hoh3038
	ERα (3ERT)	−34.4354/−13.5556	Hydrophobic interaction	Ala350, Leu 384, Leu391, Leu387, Leu346
			Polar H interactions	Leu346, Arg394, Hoh2
	PR (4OAR)	−21.1074/−18.1096	Hydrophobic interaction	Met 759, Leu763, Leu718, Phe778
			Polar H interactions	Leu718, Hoh 1129, Gln725, Arg766
	mTOR (4DRH)	−46.0927/−12.4596	Hydrophobic interaction	Lys121, His71, Tyr2038, Thr2098, Trp2101, Phe2039
			Polar H interactions	Lys121, Asp68
BER	EGFR (2J6M)	−19.2199/−12.6202	Hydrophobic interaction	Val726, Ala743, Thr790, Leu844
			Polar H interactions	Lys745, Hoh3038
	ERα (3ERT)	−34.4354/−13.0723	Hydrophobic interaction	Leu387, Leu391, Met388, Leu384, Leu346, Ala350
			Polar H interactions	Arg394, Hoh2
	PR (4OAR)	−21.1074/−13.0489	Hydrophobic interaction	Leu721, Leu718, Phe778, Met801, Met756, Leu763, Met759
			Polar H interactions	Hoh1129, Gln725, Arg766
	mTOR (4DRH)	−46.0927/−15.8240	Hydrophobic interaction	Phe130, Tyr57, Ile122, Asp68, Phe67, Lys121, Tyr113
			Polar H interactions	Tyr57, Tyr113
ANG	EGFR (2J6M)	−19.2199/−12.5955	Hydrophobic interaction	Thr854, Asp855, Lys745, Leu844, Ala743, Val726
			Polar H interactions	Lys745, Hoh3038
	ERα (3ERT)	−34.4354/−12.0107	Hydrophobic interaction	Leu384, Met388, Glu353, Leu387, Ala350, Leu391
			Polar H interactions	Arg394, Hoh2
	PR (4OAR)	−21.1074/−11.6317	Hydrophobic interaction	Met759, Phe778, Leu763, Leu763, Gln725, Leu721, Gly722, Leu718
			Polar H interactions	Arg766, Hoh1129
	mTOR (4DRH)	−46.0927/−13.6447	Hydrophobic interaction	Tyr113, Val86, Ile87, Phe130, Tyr57
			Polar H interactions	Ile87
PSO	EGFR (2J6M)	−19.2199/−13.5405	Hydrophobic interaction	Thr790, Lys745, Leu844, Val726
			Polar H interactions	Hoh3038, Lys745
	ERα (3ERT)	−34.4354/−11.9384	Hydrophobic interaction	Phe404, Leu391, Leu384, Leu346, Leu387, Ala350
			Polar H interactions	Arg394, Hoh2
	PR (4OAR)	−21.1074/−14.2120	Hydrophobic interaction	Leu763, Met759, Leu721, Met801, Met756, Phe778, Leu18
			Polar H interactions	Hoh1129, Gln725, Arg766
	mTOR	−46.0927/−14.1905	Hydrophobic interaction	Tyr113, Phe67, Phe130, Asp68, Tyr57
			Polar H interactions	Tyr113, Lys121
IMP	EGFR (2J6M)	−19.2199/−13.1183	Hydrophobic interaction	Leu718, Val726, Lys745, Leu844, Thr790, Ala743
			Polar H interactions	Hoh3038, Lys745
	ERα (3ERT)	−34.4354/−11.9196	Hydrophobic interaction	Al350, Met343, Leu525, Leu349, Leu387, Leu346, Met421, Ile424, Met388, Phe404, Leu391
			Polar H interactions	Hoh2, Arg394
	PR (4OAR)	−21.1074/−12.4716	Hydrophobic interaction	Leu887, Met801, Leu797, Met756, Phe778, Leu763, Leu718, Leu721, Gly722
			Polar H interactions	Hoh1129, Arg768, Gln725
	mTOR (4DRH)	−46.0927/−16.6907	Hydrophobic interaction	Phe20139, His71, Phe67, Phe67, Lys121, Ile122
			Polar H interactions	Val726, Ala 743, Thr 790, Leu844

### *In-vitro* ERα antagonist potential of the furanocoumarins

**Figure 7:**
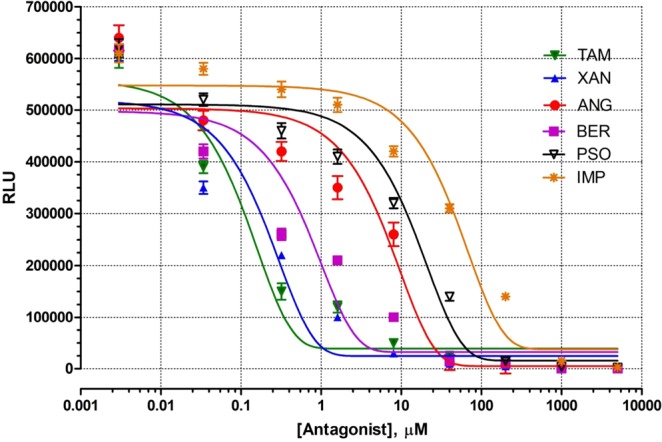
Antagonist dose response analysis of selected furanocoumarins (ANG, TAM, XAN, BER, PSO and IMP; µM) and human ERα reporter cells. Where each value is represented as mean ± SEM (n = 3). ANG: Angelicin, TAM: 4-hydroxy Tamoxifen, XAN: Xanthotoxol, BER: Bergapten, PSO: Psoralen and IMP: Isoimperatorin, ERα: Estrogen receptor.

To assess whether the chemotherapeutic potential of selected furanocoumarins is mediated via ERα receptor antagonism, they were evaluated for their antagonistic potential at various concentrations in the presence of 17β-estradiol in MCF-7 cells. Figure 7 demonstrates that the individual furanocoumarin was successful in reducing luminescence intensity (in terms of relative light units (RLU)) caused by 17β-estradiol similar to that of known antagonist TAM (positive control; IC_50_: 0.48 μM), thus indicating their ability to decrease the luciferase activity. XAN was most potent in antagonising ERα activity followed by BER, ANG, PSO, IMP. The IC50 values were 0.72 μM, 1.18 μM, 11.02 μM, 24.08 μM, and 54.32 μM for XAN, BER, ANG, PSO and IMP respectively. Thus, the results reveal the estrogen receptor dependent mechanism of the selected furanocoumarins for their therapeutic activity in MCF-7 cells.

### *In-vitro* EGFR antagonist potential of the furanocoumarins

**Figure 8:**
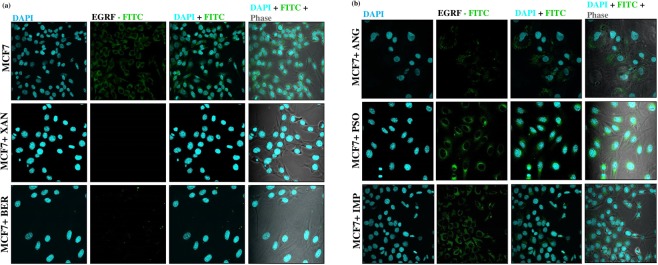
Immunofluorescence analysis of EGFR in MCF-7 cells (n = 3). DAPI: Fluorescent blue (nucleus; FITC green). EGFR expression following treatment with (**a**) XAN and BER, (**b**) ANG, PSO, IMP was indicated by its localization to the cell membrane of MCF-7 cells. For immunofluorescence staining was analysed at (x160). EGFR: Epidermal Growth Factor Receptor, ANG: Angelicin, TAM: 4-hydroxy Tamoxifen, XAN: Xanthotoxol, BER: Bergapten, PSO: Psoralen and IMP: Isoimperatorin.

To determine the antagonists (XAN, BER, ANG, PSO and IMP) mediated changes in the expression of EGFR in cell membrane of MCF-7 cells, immunofluorescence analysis was performed. The results (Fig. 8a,b) demonstrates evidently upregulated EGFR expression in MCF-7 cells which significantly decreased following treatment of the cells with the above-mentioned respective furanocoumarins. XAN was most potent in preventing localization of EGFR in membrane of the MCF-7 cells followed successively by BER, ANG, PSO, IMP, thus validating inhibition of EGFR expression as one of the therapeutic mechanisms.

### *In-vitro* mTOR inhibitory potential of the furanocoumarins

**Figure 9:**
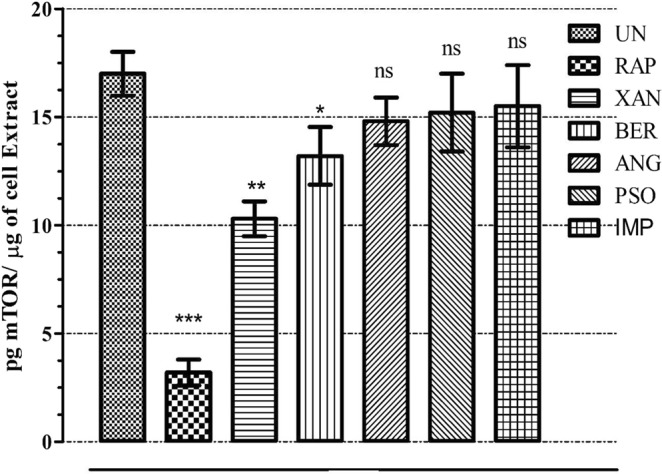
*In-vitro* mTOR inhibitory activity of the selected furanocoumarins using ELISA where each value is represented as mean ± SEM (n = 3). Comparison: RAP, XAN, BER, ANG, PSO, IMP with UN. ***p < 0.001, **p < 0.01, *p < 0.05 and ^ns^p > 0.05. UN: Untreated, RAP: Rapamycin, XAN: Xanthotoxol, BER: Bergapten, PSO: Psoralen and IMP: Isoimperatorin, mTOR: Mammalian target of Rapamycin.

In order to validate the *in-silico* studies showing high binding affinities of the furanocoumarins to mTOR, *in-vitro* ELISA assay was performed to correlate mTOR levels with their inhibitory potential. As depicted in Fig. 9, mTOR level was evidently reduced on treatment with RAP (p < 0.001) that acts as a positive control in comparison to the untreated cells. Similar to RAP activity, all the other furanocoumarins also alleviated mTOR levels with XAN showing significant decrease (p < 0.01) followed by the inhibitory activity of BER (p < 0.05). Thus, ELISA assay of mTOR confirms that the therapeutic potential of selected compounds is contributed due to mTOR inhibition as indicated from the binding affinities shown in *in-silico* studies.

## Discussion

Coumarins are a class of phytocompounds which have a benzene ring attached to a pyrone ring. The main types of coumarin classification are simple coumarins, furanocoumarins, pyranocoumarins and pyrone ring substituted coumarins. In the current study, we are focusing on furanocoumarin compounds which are five-membered furan ring compounds substituted to coumarin nucleus^26^. Psoralen and Angelicin are the two isomeric forms which are the precursors to other angular and linear furanocoumarins^27^. Furanocoumarins are compounds released by plants in stress conditions to combat against fungi, bacteria and insects^28^. Reaction with DNA of stress-inducing agents leads to disruption of replication on exposure to UV light^29^. Due to their activity against DNA replication, furanocoumarins have drawn much attention towards their use in anti-cancer therapies targeting malignant transformations^11^.

**Figure 10:**
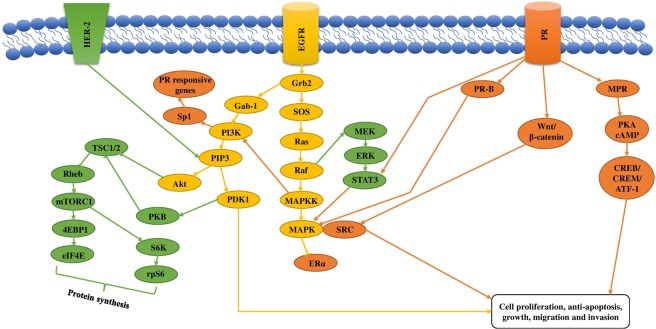
The figure shows an MPR route that gets activated by the stimulation of progesterone receptor and has a role in cell proliferation by upregulation of PKA/cAMP by the activation of CREB/CREM/ATF-1. Activation of PR also upregulates Wnt/β-catenin pathway which leads to cell proliferation and tumorigenesis by the activation of MAPK/SRC by upregulation of transcription factor, Sp1. Activation of EGFR upregulates Ras-MAPK pathway by the phosphorylation of binding domain Grb2 which has an effect on cell proliferation, anti-apoptosis and invasion. Grb2 also upregulates PIP3 by activation of Gab1, PI3K and AKT pathways that is responsible to cell invasion. mTOR complex (mTORC1) is upregulated by certain hormones and growth factors through SOS/Ras/Raf-MEK-ERK pathway or by PI3K-PDK1-PKB pathway or by both. These pathways upregulate Tuberous Sclerosis Complex (TSC1/2) which further downregulate Rheb which is a small G-protein responsible for protein synthesis by S6K-rps6.

In the current study, XAN, BER, ANG, PSO, IMP were selected on the basis of their best docking scores against breast cancer which was further validated by specific *in-vitro* assays of ERα, EGFR and mTOR. Breast cancer initiation and progression is triggered by certain cellular downstream signaling pathways which are initiated by the activation of ERα, PR, EGFR and HER-2 receptors. Some of the major downstream pathways activated in breast cancer are shown in Fig. 10. The major proteins involved are PI3k, Akt, mTOR, PKB and Wnt/β-catenin. According to reports, Angelicin inhibited breast cancer by mitochondria-dependent apoptosis by downregulation of Bcl-2 (apoptosis-regulating gene) and by the upregulation of PI3K/RAC α serine/threonine protein kinase (AKT) signaling pathway which caused breakage of DNA strands^30^. Other studies have shown that Bergapten enhanced p53 gene expression which resulted in apoptosis and a decrease in cell proliferation in breast cancer cells. The pathway involved in anti-cancer mechanism was found to be LXR/PI3K/AKT and IDOL/LDLR pathway during carcinogenesis. Bergapten has also been reported to alter the glucose and lipid metabolism causing death of breast cancer cells due to loss of counter balance^31^. Further studies showed that Psoralen downregulated Fra-1 gene which has a key role in the promotion of tumor cell proliferation, increase in cell and vascular invasion and apoptotic inhibition^32^. Psoralen also limited the activation of β-catenin which functioned as a key activator of Wnt signaling in the nucleus. Wnt/β-catenin had a major role in tumorigenesis regulation and in cell-cycle arrest at various phases^33^. IMP, a furanocoumarin found widely in umbelliferous plants was reported to show anti-cancer activity by the upregulation of Bax expression and the downregulation of Bcl-2 expression which caused apoptosis. This showed that IMP promoted the release of cytochrome C from mitochondria to cytoplasm^34^.

Furanocoumarins are abundant coumarins which are found naturally in vegetables and fruits. They are mainly common in species belonging to umbelliferae and rutaceae families. Examples of rutaceous fruits include grapefruit, lime and lemon. Juice, oils and orange flesh contain relatively lower concentrations of furanocoumarins. Examples of umbelliferous plants with furanocoumarin content are celery, carrots, carrot juice (canned and fresh), parsnip and parsley. Psoralen and Bergapten were reported to be found abundantly in celeriac, celery, parsley, dill, cumin, lemon and lemon juice, lime and lime juice, fig, parsnip, carrot and carrot juice, grapefruit and grapefruit juice. Bergapten also had its abundance in turnip, coriander, orange and orange juice. Angelicin was mostly reported to be found in parsnip. The other uncommon sources of furanocoumarins are hogweed, fennel and Saint John’s wort^11^.

Based on the binding affinities as exhibited by the docking studies supported by the *in-vitro* assays, the current study comes to the conclusion that the five selected furanocoumarin compounds i.e. XAN, BER, ANG, PSO and IMP can be considered as potent anti-breast cancer agents against ERα, PR, EGFR and mTOR. Also, the compounds can be further investigated by carrying out *in-vitro* and *in-vivo* studies on breast cancer models for the management and prevention of breast cancer.

## Methods

### Hardware and software

Docking studies were carried out in the HP Intel® Core (TM) 2 Duo CPU, processor E8300@2.83 Hz, memory (RAM) 1.00 GB, 32-bit Operating system, Windows Vista^TM^ Business using the BioSolveIT (Lead IT 2.1.3). Ligand preparation of the selected 23 furanocoumarins having anti-breast cancer potential was performed using the Lig Prep Wizard of Maestro 8.5 installed in a Dell system (3.4 GHz processor, 512 RAM, 80GB Hardisk) with Red Hat Linux Enterprise (version 3.0) as the operating system.

#### Selection of ligands and receptors

The 23 furanocoumarins that were considered for the study were obtained from PubChem (https://pubchem.ncbi.nih.gov/). The X-ray crystal structures of the receptors ERα, PR, EGFR and mTOR were retrieved from the Protein Data Bank having PDB IDs 3ERT^22^, 4OAR^23^, 2J6M^24^ and 4DRH^25^ respectively.

### Ligand preparation

All the selected molecules were drawn using 2D and 3D option of Chem. Draw Ultra 10.0.and saved in mol2 format. These molecules were imported into the project table of the Maestro 8.5, Schrodinger. Energy minimization, conformational analysis, and ligand preparation were performed using the Lig prep 2.2 application option and exported in the SDF format. Further, these molecules were imported into the docking library of FlexX 2.1.3 docking software and used for docking.

### Molecular docking studies

Based on the literature, ERα, PR, EGFR, and mTOR were selected as targets for breast cancer. The X-ray crystal structure of ERα and co-crystallized ligand (PDB ID: 3ERT), PR and co-crystallized ligand (PDB ID: 4OAR), EGFR and co-crystallized ligand (PDB ID: 2J6M), and mTOR and co-crystallized ligand (PDB ID: 4DRH) were availed from Protein Data Bank.

The possible binding modes between the ligands and the target protein 3ERT, 4OAR, 2J6M, and 4DRH were loaded in the BioSolveIT FlexX 2.1.3. FlexX is a computer program for predicting protein-ligand interactions. For a given protein and a ligand, FlexX predicts the geometry of the complex as well as an estimate for the strength of binding.

Preparation of the binding site was done using the Receptor Intelligence of the Receptor Preparation Wizard and this includes selection of chains, receptor protonation, and tautomers. The active site of the target protein was defined around a radius of 6.50 Å. FlexX uses the constructive incremental build up algorithm. For validation of the software the co-crystalized ligands were extracted and redocked into the active sites. To evaluate the quality of co-crystallized ligands, their Root Mean Square Deviation (RMSD) values were obtained. An RMSD value cut-off lesser than 2 Å is considered a good prediction for computed ligand-protein confirmation^35^. The results were compared with reference compounds obtained from the corresponding PDB IDs. The docking scores and the 2D and 3D pose views were generated for further analysis of the interactions and binding affinities of the selected 23 furanocoumarins molecules.

#### Drug likeness calculations

The compounds were checked for drug-likeness by the application of Lipinski’s rule of five by obtaining the molecular properties and bioactivity prediction from Molinspiration (http://www.molinspiration.com/). The drug-likeness was examined with the help of the following attributes: Hydrogen donors (not more than 5), hydrogen bond acceptors (not more than 10), partition coefficient (not more than 5), rotatable bonds (less than 10), total polar surface area (not more than 140) and molecular weight (less than 500 g/mol). The SMILES format of the furanocoumarin compounds was obtained from Zinc database (http://zinc.docking.org).

### *In-vitro* ERα reporter antagonist assay

The reporter study was confirmed using human estrogen receptor α (ERα) reporter assay system, 96-well format assay (Indigo Biosciences, PA, USA) as per the manufacturer’s instructions. Following primary thawing (37 °C) in water bath of cell recovery medium (CRM) and compound screening method (CSM), appropriate dilution of treatment compounds (XAN, BEG, ANG, PSO and IMP) were prepared for 17 antagonist mode screening. Then suitable dilutions of positive control (17β-estradiol) was prepared. 10 µl of the thawed CRM (37 °C) was transferred to the tubes of retrieved frozen reported cells which were then recapped and rested in water bath (5–10 minutes) resulting in cell suspension of final volume 12 ml. The tube of reporter cells was then removed from water bath and gently but repeatedly inverted for dispersion of cell aggregates and obtaining a homogenous cell suspension. Then bulk suspension of the reporter cells was suspended by 2X concentration of the reference agonist with subsequent dispersion of this cell suspension into each well of the assay plate. This was followed by dispensing respective treatment media (2 × concentration) into appropriate assay wells and then incubating (22–24 hours) the well plate in humified incubator (5% CO_2_ and 37 °C). Overnight thawed detection substrate and detection buffer (4 °C) were removed from the refrigerator and placed in a low light area for 30 minutes prior to receptor activity quantification for room temperature equilibration. After that, the tubes were gently inverted repeatedly to obtain homogenous solution while the plate reader was set to luminescence mode. Entire volume of detection buffer was transferred into detection substrate while producing 12 ml of luciferase detection reagent (LDR). After incubating for 22–24 hours, adhering cells at the well bottom were obtained by carefully discarding all media. Subsequently, LDR (100 µl) was added to each well and allowed to rest for 5 minutes at room temperature and then luminescence was quantified using Molecular Devices Luminometer Equipment-SpectraMax i3x, molecular devices, CA, USA.

### *In-vitro* mTOR inhibition assay

For the mTOR assay, MCF-7 cells were cultured in DMEM medium supplemented with 10% FBS streptomycin (100 U/ml) and penicillin (100 mg/ml) and maintained in a humified atmosphere of 5% CO_2_. The MCF-7 cells were then treated with 20 nM of Rapamycin (+ve control), XAN, BEG, ANG, PSO and IMP for 24 hours respectively^36^. The preparation of sample extracts from adherent cells was performed by direct lysis. The qualitative measurement of phosphorylated Ser2448 of mTOR protein in cell lysates was determined using mTOR pSer2448 *in-vitro* ELISA (ab168358) following manufactures instruction. Briefly, following dilution of samples in supplemented incubation buffer (1×) within working assay range, 50 μl of each sample was added to 96 well plates, sealed and incubated for 2 hours at 400 rpm at room temperature. With subsequent washing of each well thrice, 50 μl mTOR pSer2448 primary detector antibody (1×) was added to each well and incubated, shaking at room temperature for 1 hour. Again, the wells were washed and followed by incorporation of (1×) HRP labelled secondary detector antibody in (1×) incubation buffer (50 μl). With final washing (×3), HRP development solution (100 μl) was added to each well and after shaking for 30 minutes at room temperature in dark, 100 μl of stop solution was instilled into each well and end point was determined at 450 nm using microplate reader.

### Immunofluorescence analysis of EGFR

EGFR expression in MCF-7 cells was determined using anti-EGFR affibody molecule FITC (abcam) following standard protocol [25]. This anti-body acts as a ligand having specific activity against human EGFR thus binding to it. Briefly MCF-7 cells were cultured in DMEM medium supplemented with 10% FBS streptomycin (100 U/ml) and penicillin (100 mg/ml) and maintained in a humified atmosphere of 5% CO_2_. The MCF-7 cells were then treated with 20 nM XAN, BEG, ANG, PSO and IMP for 24 hours respectively. PBS was used to wash the monolayer of MCF-7 cells followed by fixation with 4% paraformaldehyde at room temperature for 10 min. The cells were stained with 2 µg/ml of anti-EGFR affibody at 37 °C for 20 min after washing. After removing the chamber, anti-fade regent with DAPI(Invitrogen) was used to mount the slides and were analyzed under a confocal microscope (FLOWVIEW, Olympus).

## Supplementary information

Supplementary information: Figure S1
