# Supplementary material for: Structure Based Multitargeted Molecular Docking Analysis of Selected Furanocoumarins against Breast Cancer
Source: Sci Rep. 2019 Oct 31;9:15743. doi: 10.1038/s41598-019-52162-0 (PMC6823401; doi:10.1038/s41598-019-52162-0)
Supplement: Supplementary file 1 — Supplementary information: Figure S1 [file 41598_2019_52162_MOESM1_ESM.pdf]

Structure Based Multitargeted Molecular Docking Analysis of Selected Furanocoumarins against Breast Cancer

Reetuparna Acharya[1], Shinu Chacko[2,4], Pritha Bose[1], Antonio Lapenna[3], Shakti Prasad Pattanayak[1*]

[1] Division of Advanced Pharmacology, Department of Pharm. Sciences & Technology, Birla Institute of Technology, Mesra, Ranchi- 835215

[2] Division of Pharmaceutical Chemistry, Department of Pharm. Sciences & Technology, Birla Institute of Technology, Mesra, Ranchi- 835215, Jharkhand

[3]Department of Oncology and Metabolism, University of Sheffield Medical School, Beech Hill Road, Sheffield S102RX, United Kingdom

[4]Research Manager, Clinical Pharmacology and Pharmacokinetics, Sun Pharmaceutical Industries Limited, Gurgaon, India-122015

***Corresponding Author:** Dr. Shakti P Pattanayak; **Contact:** +91-9123240698, 0651-2275444/896-Ext. 4832; **E-mail:** sppattanayak@bitmesra.ac.in

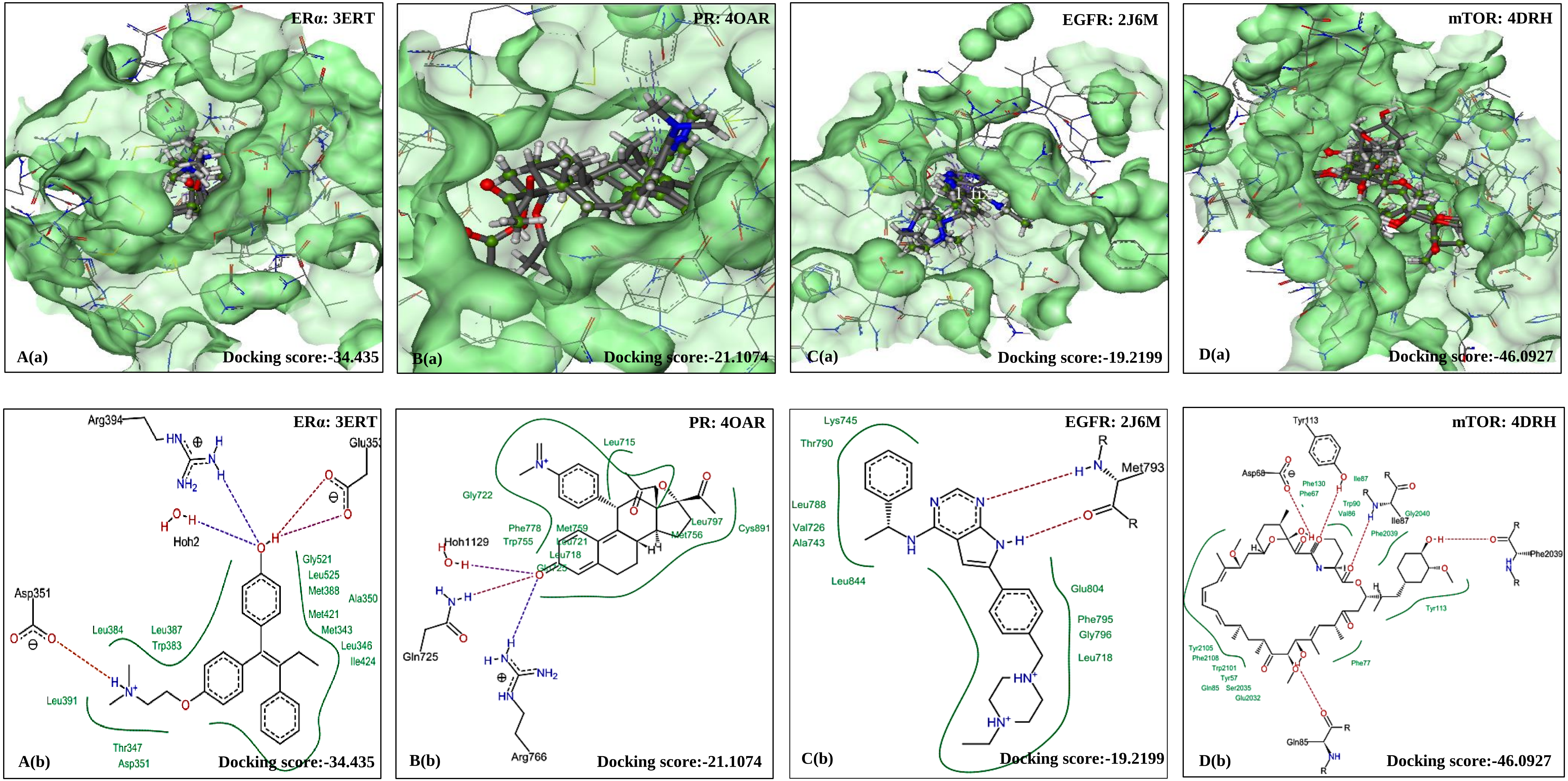

Fig. S1. Molecular docking analysis of reference compounds (a): Pose view of interaction of reference compounds with receptors ERα, PR, EGFR and mTOR. (b): Overlay of reference compounds in active pockets of ERα, PR, EGFR and mTOR. ERα: Estrogen receptor, PR: Progesterone receptor, EGFR: Epidermal growth factor receptor and mTOR: Mammalian target of Rapamycin having RMSD values 0.7766 Å, 1.1922 Å, 1.1133 Å and 1.6347 Å respectively.
